# Relative Time Compression for Slow-Motion Stimuli through Rapid Recalibration

**DOI:** 10.3389/fpsyg.2017.01195

**Published:** 2017-07-17

**Authors:** Saya Kashiwakura, Isamu Motoyoshi

**Affiliations:** ^1^Department of Integrated Sciences, The University of Tokyo Tokyo, Japan; ^2^Department of Life Sciences, The University of Tokyo Tokyo, Japan

**Keywords:** time perception, velocity, motion, duration estimation, adaptation, psychological

## Abstract

A number of psychophysical studies have shown that moving stimuli appear to last longer than static stimuli. Here, we report that the perceived duration for slow moving stimuli can be shorter than for static stimuli under specific circumstances. Observers were tested using natural movies presented at various speeds (0.0× = static, 0.25× = slow, or 1.9× = fast, relative to original speed) and indicated whether test duration was perceived as longer or shorter than comparison movies presented at their original speed. While fast movies were perceived as longer than slow and static movies (in accordance with previous studies), we found that slow movies were perceived as shorter (i.e., time compressed) compared to static images. Similar results were obtained for artificial stimuli consisting of drifting gratings. However, time compression for slow stimuli disappeared if comparison stimuli were replaced by a white static disk that removed repetitive exposures to moving stimuli. Results suggest that duration estimation is modulated by contextual effects induced by the specific diet – or distribution – of prior visual stimuli to which observers are exposed. A simple model, which includes a rapid recalibration of human time estimation via adaptation to preceding stimuli, succeeds in reproducing our experimental data.

## Introduction

We are able to judge the duration of an external event, but our performance is not always constant. It has been shown that human estimation of event duration depends on a variety of factors including temporal contexts (Jazayeri and Shadlen, 2010; Cicchini et al., 2012), adaptation (Johnston et al., 2006, 2008; Burr et al., 2007; Ayhan et al., 2009, 2011; Bruno and Johnston, 2010; Bruno et al., 2010, 2013; Bruno and Cicchini, 2016), spatial configuration (Gorea and Kim, 2015; Aoki et al., 2016), and internal states such as attention (Tse et al., 2004; Cicchini and Morrone, 2009) and emotion (Stetson et al., 2007). Above all, however, one of the main factors widely reported in psychophysical literatures as influencing duration estimation is the amount of change contained in the target event itself (Fraisse, 1963; Poynter, 1989; Brown, 1995; Kanai et al., 2006; Eagleman, 2008; Kaneko and Murakami, 2009).

A number of studies have shown that stimuli containing more changes are perceived to last longer than stimuli containing fewer changes (e.g., Brown, 1995; Kanai et al., 2006; Kaneko and Murakami, 2009). Stimulus changes that can induce time dilation include changes in speed (Kaneko and Murakami, 2009; Yamamoto and Miura, 2012), temporal frequency (Kanai et al., 2006), and apparent distance (Gorea and Hau, 2013). The large body of evidence that more changes lead to time dilation is currently reflected in fundamental assumptions underlying models of time perception. For instance, the classical “internal clock” model suggests that the perceived duration of a stimulus is the result of the accumulated number of pulses generated by a pacemaker – that is, a stimulus undergoing more changes speeds up the pacemaker rate and is therefore perceived to last longer (Creelman, 1962; Treisman, 1963; Treisman et al., 1990).

According to current models of time perception and their supporting evidence, any dynamic stimulus (even an extremely slow-moving stimulus) must always be perceived longer than a static stimulus involving no change at all. In this brief report, however, we show that a natural movie presented at unnaturally slow speeds is perceived to last for very short durations if observers compare it to a movie presented at its original natural speed. Perhaps most noteworthy is that perceived duration for a slow movie is shorter than perceived duration for a static image of the same physical duration. We observed this time compression phenomenon not only for natural movies but also for artificial stimuli such as sinusoidal gratings. Interestingly, time compression diminished if the comparison movie was replaced by a static disk. These results led us to hypothesize that time perception undergoes recalibration via adaptation to the distribution of prior stimuli to which observers are exposed. A simple model that incorporates contextual effects induced by preceding stimuli successfully replicated all our experimental data. Together, our findings suggest that human time estimation is adjusted rapidly and imply that previously reported effects in time perception might also be the product of such a recalibration process.

## Experiment 1

### Methods

#### Apparatus

Visual stimuli were generated on a graphics card (NVIDIA Quadro 2000) hosted by a computer (DELL Precision T1650) and displayed on a LCD monitor (BenQ XL2430T) with a refresh rate of 60 Hz. The pixel resolution of the CRT was 0.016 deg/pixel at the viewing distance of 100 cm we used. The experiment was done in a dark room.

#### Observers

Six naïve paid volunteers and one of the authors (SK) served as observers. Observers were 21.5 years old on average (20–24 years old) and had normal or corrected-to-normal vision. All the experiments were approved by the research ethics committee at the University of Tokyo and consent forms were duly completed.

#### Stimuli

**FIGURE 1:**
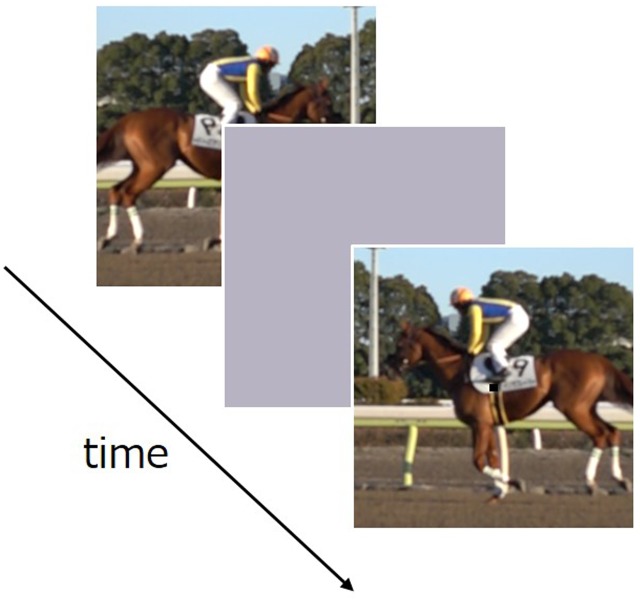
Schematic of the stimulus sequence in Experiment 1.

Visual stimuli consisted of a natural movie (a running horse, 4.1 degree × 4.1 degree, **Figure 1**) taken by a high-speed video camera (Sony DSC-RX10M2, 960 fps). Gamma correction was applied to the stimulus on the assumption that the gamma characteristic of the camera was 2.0. All stimuli were presented in the center of the display’s uniform gray background of 45 cd/m^2^. A small black fixation point was shown throughout the experiment in the center.

#### Procedure

We measured apparent movie duration via a two-alternative forced choice method (2AFC). In each trial, test stimuli and comparison stimuli were sequentially displayed with an inter-stimulus intervals (ISI) ranging from 1.0 to 2.0 s. Test stimuli were presented either at 0.0× (static), 0.25× (slow), or 1.9× (fast) relative to the movie’s original speed. Comparison stimuli were presented at the original speed with a duration of either 0.5, 1.1, or 1.6 s.

The physical duration of the test was varied in accordance with a staircase method described below. Presentation order was randomized across trials. Observers viewed the fixation point and reported which of the two stimuli appeared to last longer by pressing one of two buttons. Observers were instructed to avoid using unnatural strategies such as counting time as much as possible. Subsequent trials started 1.0 s after the observers’ response. To minimize motion aftereffects, the first stimulus of each trial was always flipped along the vertical axis while the second stimulus was not.

Test stimulus duration was varied in accordance with a staircase method. The duration of the test was either lengthened or shortened by one step depending on whether the observer reported that the comparison was longer or shorter than the test, respectively. The step size was chosen from a range of 0.07–0.53 s based on pilot experiments for each observer and for each physical duration of the comparison. The step size was doubled until the first reversal. In each measurement session, two staircases for each stimulus condition were randomly interleaved, and each staircase terminated when the number of trials after the first reversal exceeded 5. Each observer ran at least four sessions, and the point of subjective equality (PSE) was estimated by a maximum likelihood method. The apparent duration of each stimulus condition was defined as the product of the physical duration of the comparison and the ratio of the duration of the comparison to PSE. We also calculated the just-noticeable difference (JND) of the duration between test and comparison as a measure of the sensitivity for discriminating duration.

### Results

**FIGURE 2:**
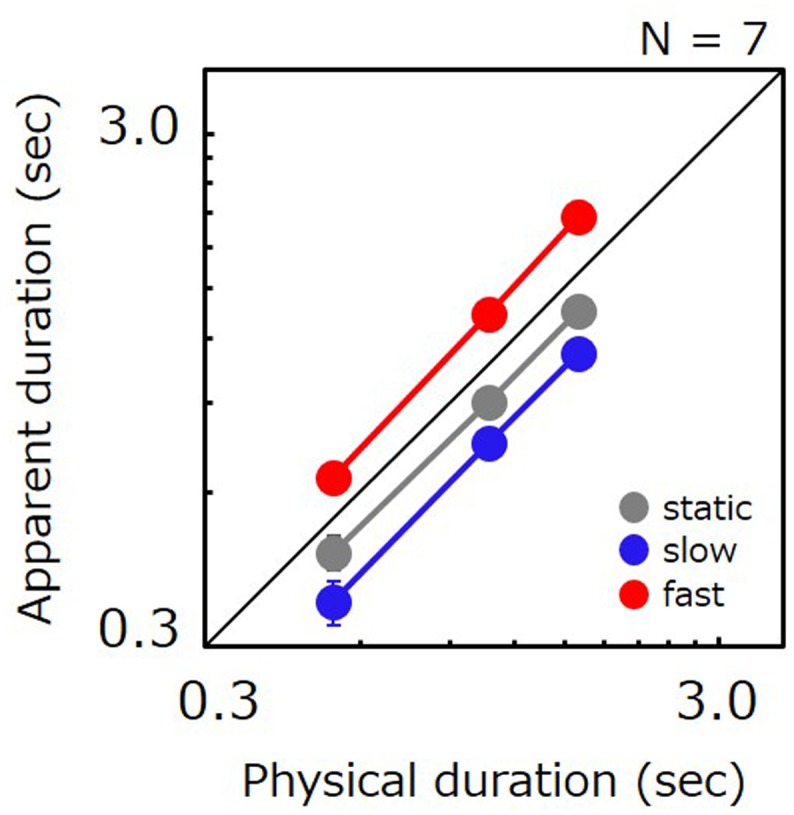
Apparent duration of a natural movie as a function of physical duration. Gray, blue, and red circle indicate the results for the static, slow, and fast stimuli, respectively. Error bars indicate SE across the observers.

**Figure 2** shows the apparent duration for each tested condition as a function of the comparison’s physical duration. In agreement with previous studies, perceived duration for fast stimuli was 1.54 times longer than for static stimuli. However, perceived duration for slow stimuli was shorter than for static stimuli (0.83 times). Ratios of perceived-to-physical duration were submitted to a two-way repeated measures ANOVA with factors of duration and speed. The analysis revealed a significant main effect of speed [*F*(12,2) = 26.082, *p* < 0.001], no significant main effect of duration [*F*(12,2) = 0.116, *p* = 0.892], and no significant interaction [*F*(24,4) = 0.339, *p* = 0.849]. *Post hoc* comparisons revealed significant differences between static vs. slow (*t* = 2.794, *p* = 0.016), static vs. fast (*t* = 4.371, *p* < 0.001), and slow vs. fast (*t* = 7.165, *p* < 0.001). We have also carried out a non-parametric analysis in which we directly compared the apparent duration of slow stimuli with that of static stimuli for individual data points. The analysis revealed that the PSE for slow stimuli was shorter than for static stimuli for 19 out of 21 data points in total, thereby indicating that slow movies were perceived as shorter than static images for almost all conditions and all observers.

A repeated measures ANOVA performed on JNDs revealed a significant main effect of speed [*F*(12,2) = 5.139, *p* = 0.024], duration [*F*(12,2) = 16.911, *p* < 0.001], and no significant interaction [*F*(24,4) = 1.461, *p* = 0.245]. *Post hoc* comparisons revealed significant differences between slow vs. fast (*t* = 3.131, *p* = 0.009), 0.5 s vs. 1.6 s (*t* = 5.714, *p* < 0.001), and 1.1 s vs. 1.6 s (*t* = 3.794, *p* = 0.003), but not for static vs. slow (*t* = 0.969, *p* = 0.352), static vs. fast (*t* = 2.162, *p* = 0.052), and 0.5 s vs. 1.1 s (*t* = 1.921, *p* = 0.079). The lack of a significant difference between the static vs. slow implies that time compression for slow stimuli cannot be attributed to potential artifacts whereby task difficulty would have depended on speed.

These results illustrate that slow stimuli can be perceived as shorter than static. This fact is clearly contradictory to the conventional results that the apparent duration of a visual stimulus increases with its speed. However, considering that stimuli commonly used in previous studies were artificial (i.e., gratings), it could be that the time compression observed for slow stimuli is attributable simply to the use of natural movies. To examine this possibility, we employed artificial stimuli – luminance sinusoidal gratings – in the subsequent experiment to see whether time compression is still manifest. Indeed, observing time compression with artificial gratings would generalize the phenomenon to a larger set of moving visual stimuli. By contrast, failure to observe time compression with gratings would imply that the phenomenon is specific either to natural movies or even perhaps tied uniquely to the movie of a running horse we have used in Experiment 1.

## Experiment 2

We measured the apparent duration for artificial stimuli, namely luminance sinusoidal gratings presented at various speeds, using the same methods as in Experiment 1.

### Methods

**FIGURE 3:**
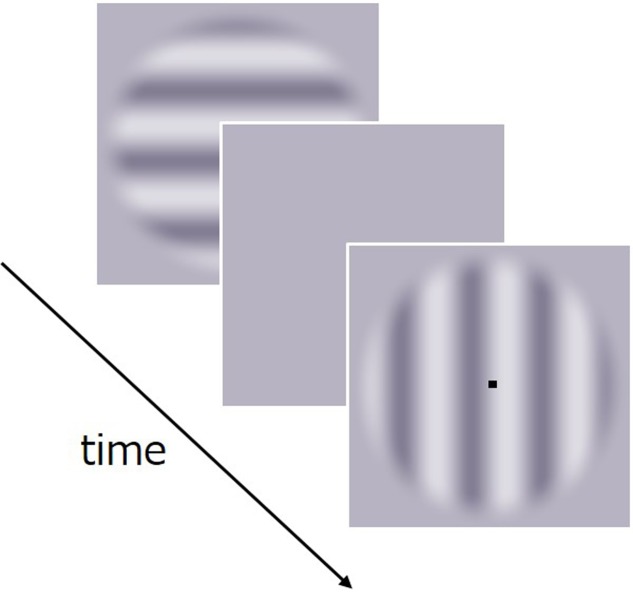
Schematic of the stimulus sequence in Experiment 2.

The paradigm used in Experiment 2 was identical to that in Experiment 1 with the following exceptions. Stimuli consisted of a drifting luminance sinusoidal carrier grating within a circular window tapered by a cosine wave of 0.5 deg wavelength (**Figure 3**). The carrier’s spatial frequency was 1.0 c/deg, Michelson contrast was 50%, and mean luminance was 45 cd/m^2^. Test stimuli drifted either at 0, 0.5, or 7.5 Hz. The temporal frequency of comparison stimuli was arbitrarily set to 3.8 Hz given that a natural “original” speed for drifting grating does not apply. The original natural movie had a 1/f temporal frequency spectrum as many natural movies generally do. When the spectrum was calculated for a limited spatial frequency band around that of the grating (1.0 c/deg), it was found to peak at a range from 0 to 5 Hz, which includes the frequency of the grating (3.8 Hz).

In previous studies (e.g., Kaneko and Murakami, 2009; Bruno et al., 2012), the orientation of the test and comparison gratings remained the same. In preliminary experiments, however, we observed that static gratings presented after moving stimuli were clearly perceived to be moving due to a motion aftereffect (MAE). This observation raises the concern that, in theory, MAE artifacts could partially account for the phenomenon that moving stimuli appear to last longer. To minimize the influence of motion aftereffects and to ensure that a static stimulus remained perceptually static, we used a horizontal grating as the first stimulus in each trial and a vertical grating as the second stimulus. Seven naïve paid volunteers and one of the authors (SK) served as observers (average age of 21.6 years, range of 21–24 years).

### Results

**FIGURE 4:**
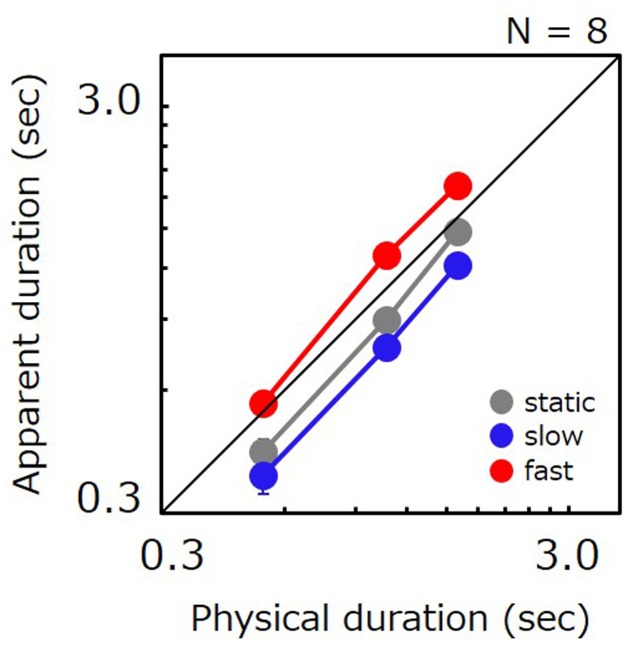
Apparent duration of a grating as a function of physical duration. Gray, blue, and red circle indicate the results for the static stimulus, the grating drifting at 0.5 Hz, and the grating drifting at 7.5 Hz, respectively. Error bars indicate SE across the observers.

**Figure 4** plots apparent test duration as a function of the comparison’s physical duration. As in Experiment 1, apparent duration for slow stimuli was shorter than for static stimuli (87%), and apparent duration for fast stimuli was longer than for static stimuli (139%). The ratio of perceived-to-physical duration was submitted to a two-way repeated measures ANOVA with factors of duration and speed. The analysis revealed a significant main effect of speed [*F*(14,2) = 24.703, *p* < 0.001], a significant difference for static vs. slow (*t* = 2.608, *p* = 0.021), static vs. fast (*t* = 4.349, *p* < 0.001) and slow vs. fast (*t* = 6.957, *p* < 0.001). The main effect of duration was also significant [*F*(14,2) = 9.755, *p* = 0.002], and a significant difference was found for 0.5 s vs. 1.1 s (*t* = 2.665, *p* = 0.018) and 0.5 s vs. 1.6 s (*t* = 4.383, *p* < 0.001). The interaction was not significant [*F*(28,4) = 0.478, *p* = 0.752]. A non-parametric analysis also revealed that the PSE for slow gratings was shorter than for static gratings for 19 out of total 24 data points.

A repeated measures ANOVA performed on JNDs revealed a significant main effect of speed [*F*(14,2) = 16.386, *p* < 0.001], duration [*F*(14,2) = 26.389, *p* < 0.001], and no significant interaction [*F*(28,4) = 2.100, *p* = 0.108]. A significant difference was found for static vs. fast (*t* = 3.680, *p* = 0.002), slow vs. fast (*t* = 5.638, *p* < 0.001), and all the pairs in three durations (0.5 s vs. 1.1 s, *t* = 3.297, *p* = 0.005; 0.5 s vs. 1.6 s, *t* = 7.255, *p* < 0.001; 1.1 s vs. 1.6 s, *t* = 3.958, *p* = 0.001), but not for static vs. slow (*t* = 1.958, *p* = 0.070).

The pattern of the results remained the same as in Experiment 1, namely that the apparent duration of slow stimuli is the shortest, followed by the static and the fast stimuli in that order. These findings indicate that time compression for slow stimuli is not restricted to natural movies but is instead generally observable in moving visual stimuli. However, since the difference for static vs. slow was relatively more profound for natural movies, it is not out of the question that natural movies may contribute a distinct component to the phenomenon.

## Experiment 3

In Experiments 1 and 2, we measured apparent duration for natural movies and grating stimuli and found that, contrary to general expectations, slow stimuli were perceived as shorter than static stimuli. These findings are unexpected because, according to prevailing models, slow moving stimuli (by definition) undergo more changes per unit time than static stimuli and should therefore induce time dilation. Here, however, we reason that an important factor that may account for our unusual results is the nature of the comparison stimulus itself. In previous studies on duration perception, static stimuli were commonly used as the comparison interval in two-alternative forced-choice methods. In contrast, our Experiments 1 and 2 employed *moving* comparison stimuli whose speeds were chosen from the bracket of speeds defined by slow and fast test stimuli. It is therefore possible that the time compression observed in our experiments is attributable to the use of a moving comparison.

To test for the above possibility, we designed a new experiment (Experiment 3) in which we emulated previous studies and used a *static* stimulus as the comparison instead of a moving one. If a moving comparison imposes a contextual effect that plays an important role in duration estimation, then time compression should be weakened or altogether eliminated if a static stimulus is used as the comparison instead. To this end, we used a white static disk as the comparison since a comparison stimulus that differs significantly from the test should minimize contextual effects that could interact with mechanisms involved in estimating test stimulus duration but should nonetheless provide an adequate static reference against which time duration can be perceptually estimated.

### Methods

**FIGURE 5:**
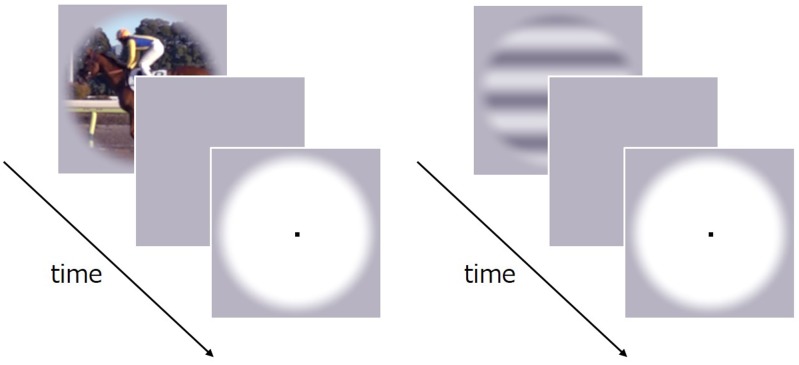
Schematic of the stimulus sequence in Experiment 3.

The paradigm used in Experiment 3 was identical to that in Experiment 1 with the following exceptions. Test stimuli consisted of either natural movies or drifting gratings used in Experiments 1 and 2. Comparison stimuli consisted of a white static disk designed to minimize contextual effects that could interfere with the processing of subsequent dynamic test stimuli (**Figure 5**). Natural movies were presented inside a 4.1-deg-diameter circle so that test stimuli were equal in size to white-disk comparisons. The duration of the comparison stimulus was varied in accordance with the staircase method. Four naïve paid volunteers and one author (SK) served as observers (average of 21.2 years, range of 20–22 years) in the experiment using natural movies, and four naïve paid volunteers and one author (SK) served as observers (average 21.4 years, range 20–24 years) in the experiment using drifting gratings.

### Result

**FIGURE 6:**
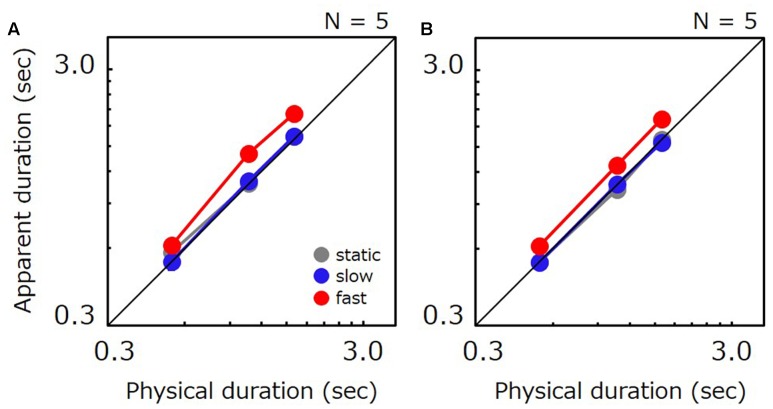
**(A)** Apparent duration of a natural movie as a function of physical duration in Experiment 3. Gray, blue, and red circle indicate the results for the static, slow, and fast stimulus, respectively. **(B)** Apparent duration of a grating as a function of physical duration in Experiment 3. Gray, blue, and red circle indicate the results for the static stimulus, the grating drifting at 0.5 Hz, and the grating drifting at 7.5 Hz, respectively. Error bars indicate SE across the observers.

**Figures 6A,B** show apparent test duration as a function of the physical duration for natural-movie and drifting-grating conditions, respectively. As in Experiments 1 and 2, fast stimuli were perceived to last longest. However, the time compression for slow stimuli which we observed in Experiments 1 and 2 has completely disappeared in Experiment 3, as no substantial difference was found between slow and static stimuli. For natural movies, fast stimuli were perceived to last longer than static ones by 21% on average. Slow stimuli were perceived to last almost the same as static ones, being shorter than the static by 3% on average. For drifting gratings, fast stimuli were perceived to last longer than static ones by 21% on average. Slow stimuli were perceived to last almost the same as static ones, being longer than the static by 1% on average. Ratio of apparent-to-physical duration were submitted to a two-way repeated measures ANOVA with factors of duration and speed. For natural movies, the analysis revealed a significant main effect of speed [*F*(8,2) = 5.867, *p* = 0.027] and a significant difference for static vs. fast (*t* = 2.797, *p* = 0.023) and slow vs. fast (*t* = 3.111, *p* = 0.014). No significant difference was observed for static vs. slow (*t* = 0.314, *p* = 0.761). No significant main effect of duration [*F*(8,2) = 0.244, *p* = 0.789] and no significant interaction [*F*(16,4) = 1.831, *p* = 0.172] were observed. For drifting gratings, the analysis revealed a significant main effect of speed [*F*(8,2) = 18.253, *p* = 0.001] and a significant difference for static vs. fast (*t* = 5.260, *p* < 0.001) and for slow vs. fast (*t* = 5.205, *p* < 0.001). No significant difference was observed for static vs. slow (*t* = 0.054, *p* = 0.958). No significant main effect of duration [*F*(8,2) = 0.009, *p* = 0.991] and no significant interactions [*F*(16,4) = 0.317, *p* = 0.862] were observed.

For natural movies, a repeated measures ANOVA performed on JNDs revealed a significant main effect of duration [*F*(8,2) = 28.397, *p* < 0.001], no significant main effect of speed [*F*(8,2) = 1.051, *p* = 0.393], and no significant interaction [*F*(16,4) = 1.652, *p* = 0.210]. A significant difference was found for all the pairs in three durations (0.5 s vs. 1.1 s, *t* = 3.572, *p* = 0.007; 0.5 s vs. 1.6 s, *t* = 7.533, *p* < 0.001; 1.1 s vs. 1.6 s, *t* = 3.961, *p* = 0.004). For drifting gratings, we found a significant main effect of duration [*F*(8,2) = 18.648, *p* = 0.001], no significant main effect of speed [*F*(8,2) = 3.409, *p* = 0.085], and no significant interaction [*F*(16,4) = 0.994, *p* = 0.439]. A significant difference was found for all the pairs in three durations (0.5 s vs. 1.1 s, *t* = 3.023, *p* = 0.016; 0.5 s vs. 1.6 s, *t* = 6.107, *p* < 0.001; 1.1 s vs. 1.6 s, *t* = 3.084, *p* = 0.015).

Whereas Experiments 1 and 2 used moving comparison stimuli, Experiment 3 used a white static disk that, by virtue of its spatial and temporal properties, we assume, has a minimal effect on the processing of subsequent stimuli. Correspondingly, Experiments 1 and 2 revealed significant differences between the apparent duration of static and slow stimuli whereas the phenomenon was absent in Experiment 3. This pattern of results is consistent with the notion that time compression for slow moving stimuli is a phenomenon that depends on the sequence of stimulus speed.

## Discussion

We measured apparent duration for natural movies and artificial gratings presented at various speeds and found that, if compared to a moving stimulus, a slow stimulus was perceived as shorter than a static stimulus (Experiments 1 and 2). This “time compression” phenomenon is inconsistent with a fundamental assumption in perceptual temporal-duration research that stimuli undergoing more change per unit time are perceived to last longer. Moreover, we found that replacing the moving comparison stimuli with a static white disk weakened and even eliminated time compression for slow moving tests (Experiment 3). Crucially, in both types of the experiments, test stimuli were identical but comparison stimuli were different. In Experiments 1 and 2, observers were frequently exposed to moving stimuli by virtue of the fact that both test and comparison stimuli involved similar speed regimes. In Experiment 3, by contrast, comparison stimuli were static, and moving stimuli were therefore presented less frequently. Given that Experiments 1 and 2 used moving comparison stimuli, it is reasonable to assume that moving comparisons play an important role in the phenomenon of time compression.

### Contextual Effects on Time Perception

Previous studies have shown that time perception is influenced by stimuli presented prior to the test stimulus. For example, apparent duration is well known to be systematically affected by adaptation to a moving stimulus (e.g., Johnston et al., 2006; Bruno et al., 2013) or to stimulus duration (Heron et al., 2012). It has also been shown that apparent duration exhibits a systematic regression toward the mean of prior stimuli (Jazayeri and Shadlen, 2010; Cicchini et al., 2012). Among these contextual factors, we explore the possibility that time compression effects observed in our experiments are partially explained by adaptation to moving stimuli. Motion adaptation is known to have differential effects on apparent test duration depending on whether the test is static or dynamic. If the test is a moving stimulus, adaptation leads to a compression in apparent duration (Johnston et al., 2006; Bruno et al., 2013). By contrast, if the test is static, adaptation leads to an opposite result – a dilation in apparent duration (Droit-Volet and Wearden, 2002; Ortega et al., 2012).

Under the assumption that the motion-adaptation rules described above are applicable to shortly presented adaptors, our results in Experiments 1–3 could be explained as follows. In Experiments 1 and 2, test stimuli were frequently preceded by moving stimuli acting as adaptors that compress the perceived duration of moving tests and dilate the duration of static tests. As the net result, slow tests were therefore perceived as shorter than static tests. In Experiment 3, however, comparison stimuli were static and, given that the frequency of observing moving stimuli was relatively low, we assume that the contextual effects were negligible. Thus, under the conditions of Experiment 3, the perceived duration of slow stimuli was not significantly below that of static stimuli.

### Rapid Recalibration by Prior Stimuli

To verify that contextual effects induced by moving stimuli can explain results from the present study, we conducted a numerical simulation using a simple model. The model makes three assumptions. First, a moving stimulus presented immediately after another moving stimulus is perceived as shorter (time compression) than if no preceding stimulus had acted as an adaptor. We assumed that only fast- and mid-speed stimuli give rise to a contextual effect given that slow comparison stimuli (natural movies: 0.25 speed, gratings: 0.5 Hz) are perceptually similar to static stimuli. Second, we assumed that a static stimulus presented immediately after a fast- or mid-speed stimulus is perceived to last longer (time dilation) than if no preceding stimulus had acted as an adaptor. Third, we assumed a conventional rate-of-change bias that a faster stimulus is perceived to last longer.

For each condition (duration × speed), +1 point was given to events corresponding to the first assumption, and -1 point to events corresponding to the second assumption. The average score was calculated within each condition, and we took that score as our indication of the quantitative shift in the internal representation of test duration. We performed simulations on actual trials that observers experienced during the experiments and calculated the average shift across observers. To account for the conventional rate-of-change phenomenon that faster stimuli are perceived as longer, we included multiplicative biases to the perceived duration of static, slow, and fast stimuli (1.0×, 1.013×, and 1.2×, respectively) relative to physical stimulus duration. The ratio for fast stimuli was estimated based on the data in Experiment 3. The ratio for slow stimuli was then defined such that the amount of dilation would be proportional to the speed of stimulus used in Experiment 2. In the end, the apparent duration of each tested condition was calculated as (physical duration) × (conventional bias) × 10ˆ (average shift × weight). The weight of the average shift was set to 0.04 so that estimated contextual effects would correspond to approximately 10% of original stimulus duration, in line with the amount reported by previous studies (Johnston et al., 2006; Ortega et al., 2012; Bruno et al., 2013).

**FIGURE 7:**
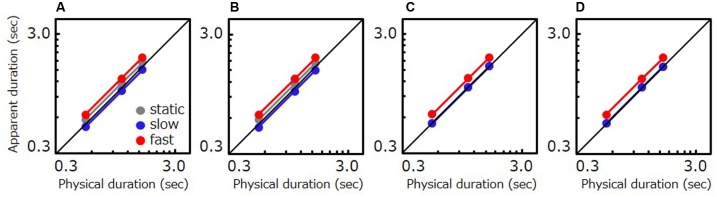
Simulated data based on the model including contextual effects. Apparent duration of a stimulus as a function of physical duration. **(A)** The result for Experiment 1 (natural movie, moving reference). **(B)** The result for Experiment 2 (artificial grating, moving reference). **(C)** The result for a natural movie in Experiment 3 (static reference). **(D)** The result a grating in Experiment 3 (static reference). Gray, blue, and red circle indicate the results for the static, slow, and fast stimulus, respectively. For **(C,D)**, the gray circles are invisible as the gray and the blue circle are almost overlapped.

As **Figure 7** shows, the model simulation in each condition qualitatively reproduces the pattern of results for human observers. The simulation illustrates that time compression for slow speeds is attributable to contextual effects induced by preceding moving stimuli. Similar patterns of simulated results were obtained over a wide range of conventional rate-of-change fast-stimulus biases (1.15–1.6) under conditions where either the bias of slow stimuli was estimated in the way described above or the weight of the average shift amount was varied from 0.03 to 0.06.

The third assumption in the model which contains a conventional rate-of-change bias was necessary to reproduce our data. Without this bias, the apparent duration simulated for fast tests was shorter than for static tests. However, only one assumption among the first two (irrespective of which) was necessary to reproduce the data – that is, similar patterns could be reproduced with either assumption alone. Manifestly, further examination is needed to further characterize the role of contextual effects in perceived stimulus duration.

In the present study, stimuli were presented in foveal vision so that observers could understand the content of a natural movie used in Experiments 1 and 3. In comparison, stimuli in previous studies were generally presented in peripheral vision. One might argue that time compression for slow stimuli reported in this study is a special case that can only be observed in foveal vision. However, time compression was not observed for slow stimuli in Experiment 3 in which stimuli were presented in foveal vision. Although we do not rule out the possibility that stimulus properties such as eccentricity may have some impact on time compression, Experiment 3 rules out that eccentricity alone accounts for time compression. We also do not rule out the possibility that speed could produce time compression by itself. In fact, individual data in Experiment 3 even show that some observers perceived slow stimuli as shorter than static stimuli.

It has been shown that adaptation-induced time compression is specific to spatial location in the retinotopic (Bruno et al., 2010; Johnston et al., 2011) and/or spatiotopic coordinates (Burr et al., 2007, 2011; Morrone et al., 2010; Bruno and Cicchini, 2016; Latimer and Curran, 2016). These findings point a possibility that the contextual effects found in the present study would also depend on the congruency in spatial location between stimuli. This possibility could be directly tested in future investigations.

One might argue that the reason why there was no difference between the static and the slow in Experiment 3 was because contrast adaptation to white static disk comparison reduced the apparent speed in slow stimuli. As contrast adaptation is tightly selective for the spatial frequency (Blakemore and Campbell, 1969; Blakemore et al., 1973; Georgeson, 1985), however, it is unlikely that a significant contrast adaptation on the gratings was induced by the static disk which had quite a different spatial-frequency component.Since natural movies presented at different speeds have different contents (e.g., the number of repeated movements of horse’s legs), one might consider that such higher-order factors played a significant role to induce unusual time compression phenomenon in slowly moving stimuli. However, since there was no difference from the results in artificial gratings (Experiment 2), we were not able to find evidence which supports the existence of a bias induced by differences in contexts.

Although the differences between the two results (Experiments 1 and 2) did not reach a significant level, time compression with slow stimuli was relatively more profound for natural movies (Experiment 1) than for drifting gratings (Experiment 2). As we demonstrated herein, contextual effects could impact duration perception; it is therefore likely that time compression was less profound in grating conditions simply because contextual effects were weakened by the alternation of both orientation and motion direction on every stimulus presentation. The other intriguing possibility is that natural movies contain distinctive information specific to natural movements such as biological motion and gravitational fall (Carrozzo et al., 2010; Lacquaniti et al., 2015). In a pioneering study, Eagleman (2004) has shown that a small flash superimposed on a slow natural movie was perceived to be ∼30% shorter than if the flash was superimposed on a movie playing at a natural speed. Eagleman (2004) interpreted this time compression as a phenomenon resulting from an efficient coding strategy that minimizes error between predictions from an internal model of Newtonian physics and sensory feedback (Eagleman, 2004; Eagleman et al., 2005; Eagleman and Pariyadath, 2009). According to this efficient-coding model, the existence of the standard speed is essential to produce time compression. That is, perhaps time compression is more profound for natural movies because the natural speed in the internal model was present only in natural movies and not in artificial drifting gratings. In addition to a partial interpretation of the results in our experiments, Eagleman’s prediction-error theory can also describe the ensemble of our results without contradicting our rapid recalibration account. Given that the standard speed in the model is established by prior input exposure, repeated exposures to a particular speed could also lead to an establishment of a standard speed and induce time compression in artificial gratings. Presumably, while both the Newtonian model and the flexible repeated-exposure model apply to natural movies, only the repeated-exposure model applies to artificial gratings. On that basis, one could perhaps attribute the greater time compression observed for natural movies to predictions from models that generate greater certainty.

The present study focused only on prior stimuli presented immediately before test stimuli. However, it is quite possible that, in line with prediction-error theory, contextual effects were determined by the entire history of stimuli. We examined contextual effects to a first approximation using only the simplest of models, but one could consider more elaborated models that take into quantitative account the known properties of time perception mechanisms (Herbst et al., 2013; Shi et al., 2013). It is nonetheless noteworthy that, despite considering only immediately preceding stimuli as adaptors, our simple model was able to qualitatively reproduce observed human data. We take this as evidence that our perception of event duration can be affected by immediately preceding stimuli presented for as little as a few hundred milliseconds.

One might consider that contextual effects may account for a range of phenomena reported in the literature on perceived event duration (e.g., Brown, 1995; Kanai et al., 2006; Kaneko and Murakami, 2009). While the idea is intriguing, we do not argue here that it can explain the conventional law in time perception that faster stimuli are perceived as lasting longer. In fact, in the present study, the fastest test stimulus was always perceived to last longer even in Experiment 3 where we sought to minimize contextual effects as much as possible, and it was necessary to introduce conventional rate-of-change bias to model the human data.

## Ethics Statement

This study was carried out in accordance with the recommendations of guideline on research ethics by the Japanese Psychonomic Society with written informed consent from all subjects. All subjects gave written informed consent in accordance with the Declaration of Helsinki. The protocol was approved by the research ethics committee at the University of Tokyo.

## Author Contributions

SK and IM designed the experiment, SK collected and analyzed the data. SK and IM interpreted and modeled the results. SK and IM wrote the manuscript.

## Conflict of Interest Statement

The authors declare that the research was conducted in the absence of any commercial or financial relationships that could be construed as a potential conflict of interest.
